# Harvesting Environment Mechanical Energy by Direct Current Triboelectric Nanogenerators

**DOI:** 10.1007/s40820-023-01115-4

**Published:** 2023-05-20

**Authors:** Chuncai Shan, Kaixian Li, Yuntao Cheng, Chenguo Hu

**Affiliations:** 1 School of Physics, Chongqing University, Chongqing, 400044 People’s Republic of China; 2 School of Energy and Engineering, Chongqing University, Chongqing, 400044 People’s Republic of China

**Keywords:** Triboelectric nanogenerators, Direct current, Working mechanism

## Abstract

As hundreds of millions of distributed devices appear in every corner of our lives for information collection and transmission in big data era, the biggest challenge is the energy supply for these devices and the signal transmission of sensors. Triboelectric nanogenerator (TENG) as a new energy technology meets the increasing demand of today's distributed energy supply due to its ability to convert the ambient mechanical energy into electric energy. Meanwhile, TENG can also be used as a sensing system. Direct current triboelectric nanogenerator (DC-TENG) can directly supply power to electronic devices without additional rectification. It has been one of the most important developments of TENG in recent years. Herein, we review recent progress in the novel structure designs, working mechanism and corresponding method to improve the output performance for DC-TENGs from the aspect of mechanical rectifier, tribovoltaic effect, phase control, mechanical delay switch and air-discharge. The basic theory of each mode, key merits and potential development are discussed in detail. At last, we provide a guideline for future challenges of DC-TENGs, and a strategy for improving the output performance for commercial applications.

## Highlights

- The basic theory, key merits and potential development of direct current triboelectric nanogenerator (DC-TENG) from the aspect of mechanical rectifier, tribovoltaic effect, phase control, mechanical delay switch and air-discharge are discussed in detail.
- This review provides a guideline for future challenges of DC-TENGs, and a strategy for improving the output performance for commercial applications.

## Graphical abstract

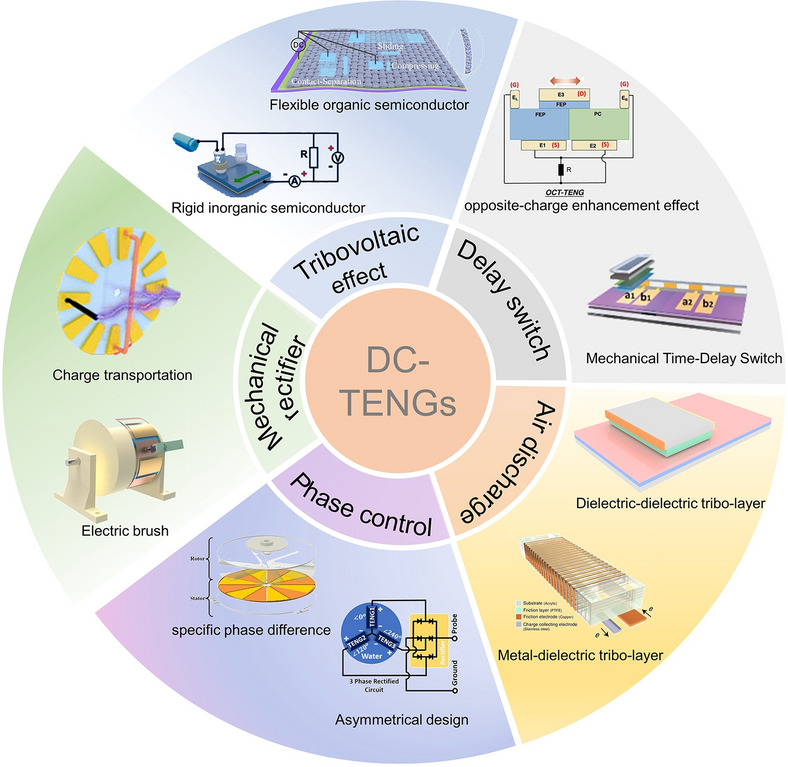

## Introduction

In the era of big data, how to meet the power supply of billions of distributed sensors is still a great challenge [1, 2]. The currently used traditional battery with limited life span is unlikely to meet the demand of such huge numbers and it is also one of the greatest threats to ecological environment. Therefore, finding out a new clean energy source technology is the most important challenge of our time [3–5]. The triboelectric phenomenon has been known for 2600 years [6, 7], but its mechanism was not clear enough to turn it into an available electric output until Wang [8] invented the triboelectric nanogenerator (TENG) in 2012. As a new energy harvesting technology, TENG is regarded as a promising energy technology in the twenty-first century because of its environmental friendliness [9, 10], lightweight [11, 12], small size [13–16], simple fabrication [17, 18], rich application scenarios [19–21]. Numerous studies have demonstrated that TENG can convert low frequency ambient energy including rain energy [22, 23], wind energy [24, 25] and water flow energy [26–28] to electric energy. Furthermore, with its self-powered characteristics, TENG exhibits significant potentials in biomedical [29, 30], artificial intelligence [31], human–machine interface [32, 33], and high voltage applications [34, 35].

TENG has two basic output modes, direct current (DC) and alternating current (AC) [36, 37]. DC TENG does not change the magnitude and direction periodically over time [38, 39]. In AC TENG, a bidirectional pulse and fixed pulse width can be obtained by reciprocating the rubbing with opposite polarity dielectric materials [40, 41]. AC TENG needs rectification to be utilized by electronic devices, which greatly limits the convenience of the device [42, 43]. Compared with DC-TENG, higher crest factor of output performance of AC TENG should be reduced referring to the energy utilization efficiency [44]. Considering the surface charge density and average power density of TENGs, many methods have been employed to improve the output performance of AC-TENG, such as charge excitation [45], space charge accumulation [40], high-k material [46] and interface lubrication [47]. However, due to inherent triboelectrification properties of the materials and the negative impact of air breakdown, the development of TENG seems to have reached a bottleneck [48].

**Fig. 1:**
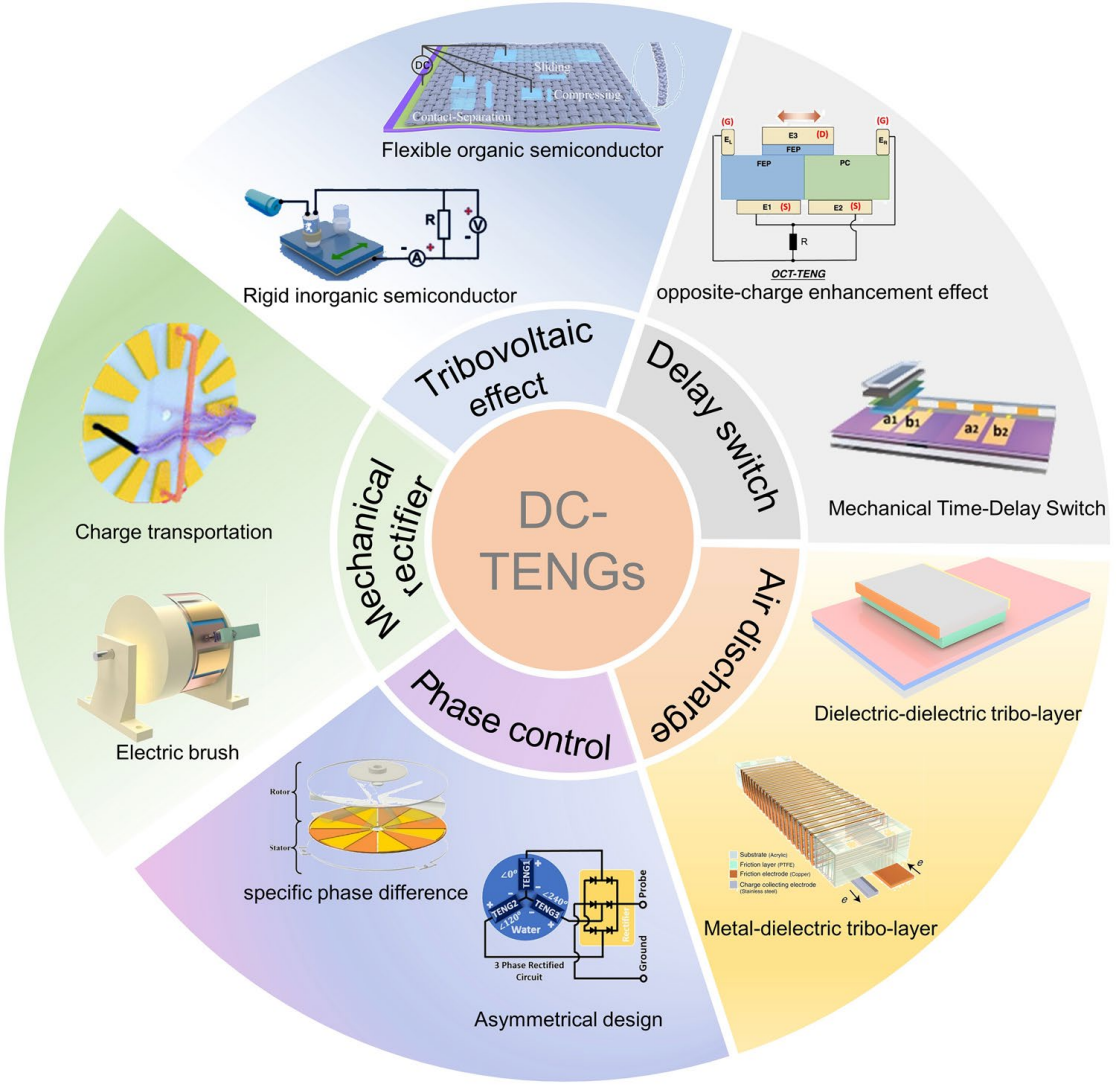
Schematic illustration of all kinds of DC-TENGs. Tribovoltaic Effect based DC-TENG, using rigid inorganic semiconductor (reproduced with permission [49]. Copyright 2020, Elsevier), and flexible organic semiconductor (reproduced with permission [50]. Copyright 2022, Royal Society of Chemistry). Delay switch based DC-TENG, using opposite-charge enhancement effect (reproduced with permission [51]. Copyright 2021, Springer Nature), and mechanical time-delay switch design (reproduced with permission [52]. Copyright 2022, Wiley–VCH). Air-discharge based DC-TENG, using metal-dielectric tribo-layer (reproduced with permission [53]. Copyright 2020, Springer Nature) and dielectric-dielectric tribo-layer (reproduced with permission [54]. Copyright 2022, Wiley–VCH). Phase control based DC-TENG, using specific phase difference (reproduced with permission [55]. Copyright 2022, Elsevier) and asymmetrical design (reproduced with permission [56]. Copyright 2018, Elsevier). Mechanical rectifier based DC-TENG, using charge unidirectional transportation (reproduced with permission [57]. Copyright 2020, Royal Society of Chemistry) and electric brush (reproduced with permission [58]. Copyright 2021, Elsevier)

To address these problems, many attempts have been carried out and DC-TENG is considered as a novel next generation TENG to generate unidirectional flow of charges in the external circuit [54]. Various DC-TENGs based on different structures and materials have been invented in recent years. Although DC TENG has made a great progress, achieving high output and high stability at the same time are still a problem to be solved. Therefore, we should clarify the category and mechanism to get a better understanding of the physics behaviors in DC TENG for the future development. Herein, we review recent progress in the novel structure designs, working mechanism and corresponding method to improve the output performance for DC-TENGs. We focus on DC TENG based on tribovotaic effect [49, 50], mechanical rectifier [57, 58], decay switch [51, 52], air-discharge [53, 54] and phase control [55, 56], as shown in Fig. 1. At last, each type of DC-TENG is highlighted by systematic comparison, and we also propose strategies for future development of DC-TENG.

## Development of DC-TENG

**Fig. 2:**
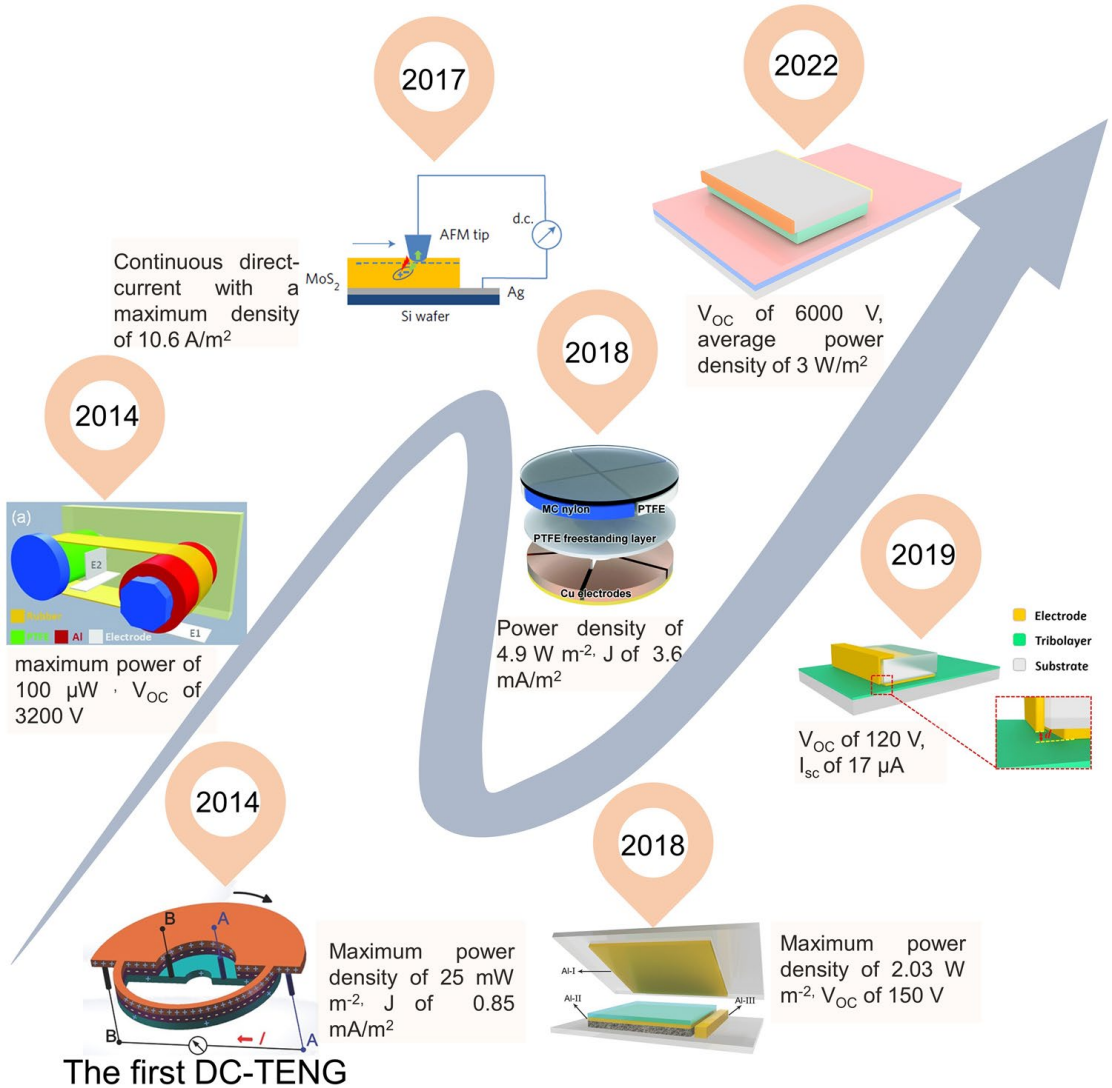
Timeline of milestones of DC-TENGs in various output modes. Reproduced with permission [59]. Copyright 2014, Wiley–VCH; reproduced with permission [60]. Copyright 2014, Wiley–VCH; reproduced with permission [61]. Copyright 2017, Springer Nature; reproduced with permission [62]. Copyright 2018, Royal Society of Chemistry; reproduced with permission [64]. Copyright 2019, Springer Nature; and reproduced with permission [54]. Copyright 2022, Wiley–VCH

Figure 2 shows representative devices in various types of DC TENG from 2014 to 2022. The first DC TENG was invented in 2014 [59], which can be used as a continuous DC source to power electronic devices without a rectifier bridge, opening a new direction in the development of TENG. Then, open-circuit voltage larger than 3200 V is achieved by rotational motion based on corona discharge in 2014 [60]. The first DC-TENG in tribovoltaic effect is developed in 2017 [61], which achieved a maximum current density of 10.6 A m^−2^. With a symmetrical design, the first phase control DC-TENG was reported in 2018 [62]. In a combination of mechanical delay switch and air breakdown induced ionized air channel, which is the first contact separation mode DC TENG developed in 2018 [63]. In 2019, a metal-dielectric tribo-layer DC-TENG was developed, which realized a constant current based air-breakdown effect [64]. Starting from the design of dielectric-dielectric tribo-layer, an average power density of 3 W m^−2^ was reported in 2022 [54]. In order to meet practical applications, DC TENG with higher output performance, optimized device structure and more durable material needs to be developed.

### Mechanical Rectifier Based DC-TENG

**Fig. 3:**
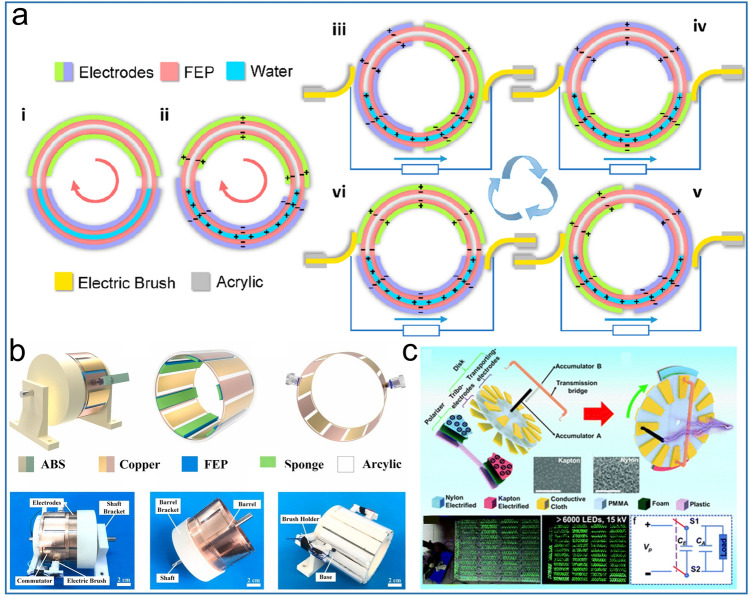
Mechanical rectifier strategy for DC-TENG. **a** Structure and working mechanism of DC-TENG with solid–liquid tribo-layer. Reproduced with permission [65]. Copyright 2019, American Chemical Society. **b** 3D structure diagram and device photograph of DC-TENG with electric brush design. Reproduced with permission [58]. Copyright 2021, Elsevier. **c** Schematic diagram of the charge unidirectional transportation DC-TENG and its application demonstration. Reproduced with permission [57]. Copyright 2020, Royal Society of Chemistry

Using flexible design of mechanical structure, AC output generated by electrostatic induction is converted into DC output to power electronic devices without diodes, which is called mechanical rectification. By employing two electric brushes on tribo-materials of opposite polarity, each electric brush is forced to transport only unidirectional charge, forming a stability DC output. Wang et al. first invented liquid–solid DC-TENG by mechanical rectification [65], including FEP tube with water in it, where the copper electrode with a ring structure and two electric brushes fixed at one end of the copper electrode. Similar to the free-standing mode, under the stimu of external forces, water as a sliding friction layer moves inside FEP tube and closely contacts it, forming a complete power generation cycle. The detailed working mechanism is shown in Fig. 3a. In the initial state, the FEP tube is negatively charged and the water is positively charged due to triboelectrification effect. When the upper electrode enters the liquid covered area and the original liquid covered area leaves the liquid, an output is produced in the external circuit. Because of the two electric brushes, the charge is transported from the upper electrode to the lower electrode through the load in the tube's rotation process, creating a steady DC output. He et al. reported an hourglass DC TENG with an electric brush [66]. Through a unique electric brush design, the PTFE and Al ball mixture during the fall form a DC signal output in the absence of a rectifier bridge. As shown in Fig. 3b, a bidirectional DC TENG is firstly proposed by Qiao et al. [58]. The TENG are divided into three parts, the mechanical structure component, the triboelectric power-generation unit, and the mechanical rectifier. As the key to producing DC output, the mechanical rectification part consists of a rolling brush and a commutator to switch current reversion. Moreover, the TENG can power calculator directly with 0.96 W m^−2^ maximum power density. TENG is known for its high voltage output, however, it is not easy to reach the high voltage in tens of kilovolts volts. Therefore, the exploration of a sustainable and stable high-voltage TENG structure has attracted attention of many researchers.

An ultrahigh voltage output direct current TENG with charge accumulation strategy is proposed by Lei et al. [57], as shown in the Fig. 3c. The TENG consists of a polarizer, disk, transmission bridge and a pair of accumulators, and with the rotation of the disk, a continuous DC output is generated. Nylon and kapton with super positive and super negative triboelectrification polarity properties are rubbed with the tribo-electrode as polarizers to create a strong electric field. Unlike the tribo-electrode, the transport electrode serves to transfer the charge induced by the polarizer to the accumulators. During the rotation of the disk, the charges induced by nylon and kapton of opposite triboelectrification polarity accumulate to the transporting-electrodes through the transport bridge. Finally, new charges continuously accumulate through the transporting-electrodes to the accumulator until the end of a period. When the transporting-electrodes and the accumulator completely coincide, the electrostatic charges are fully stored into the accumulator, reaching a high output voltage.

### Tribovoltaic Effect Based DC-TENG

Tribovoltaic effect refers to a situation when semiconductor contact with metal or semiconductor and slip relatively, electron hole pairs will be excited on the interface, thereby generating a DC output in directional separation under action of the electric field. Because conventional TENGs used insulating polymer material as the tribo-layer, their development were limited by high matching impedance and low current output. Thus, the researchers invented a type of DC-TENG based on the tribovoltaic effect using metal–semiconductor or semiconductor–semiconductor, whose structure achieved high output direct current [67–69]. According to different application scenarios, we will review this part through rigid inorganic semiconductors and flexible organic semiconductors.

#### Rigid Inorganic Semiconductors

**Fig. 4:**
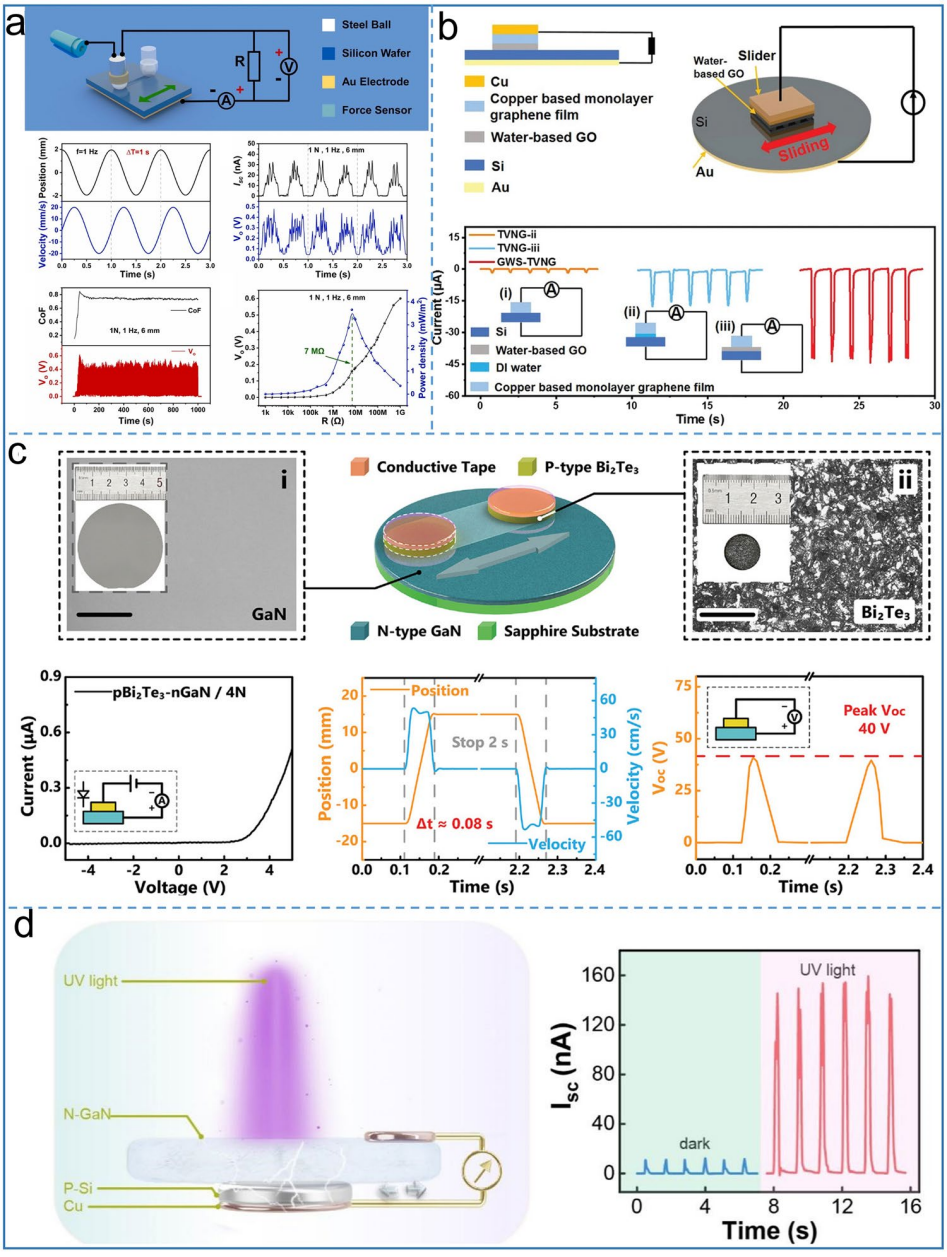
Schematic diagram of tribovoltaic effect based-DC-TENG with rigid semiconductor materials. Structure and output curves of **a** tribological-behaviour-controlled DC TENG. Reproduced with permission [49]. Copyright 2022, Elsevier. **b** Tribovotaic nanogenerator via interface sliding. Reproduced with permission [70]. Copyright 2022, Elsevier. **c** Contact-electrification-dominated TENG. Reproduced with permission [71]. Copyright 2022 Wiley–VCH. **d** TENG via Conjunction of Tribovoltaic Effect and Photovoltaic Effect. Reproduced with permission [72]. Copyright 2021, American Chemical Society

Although the tribovoltaic effect nanogenerator has a high output current density, it is difficult to operate for a long term because the output plummets due to severe wear on semiconductor materials. In recent years, several lubrication types have been reported, among which the DC-TENG proposed by Yang et al. [49]. With ball-on-flat configuration controlled performance by contact pressure, wear is effectively reduced by using polyalphaolefin SpectraSyn 4 (PAO 4) as a lubricant and achieved stable DC output, as shown in Fig. 4a. The effect of PAO 4 on the TENG was verified by the grinding depth curve. It can be seen that the wear depth of the silicon wafer ranges from 5.5 µm to 84.1 nm with the lubrication after 20,000 cycles. The water-based graphene oxide solution can also be used as a metal–semiconductor lubricant to improve output performance by improving sliding surface carriers and increase the lifetime of the TENG, which is first invented by Qiao et al. [70]. The TENG consists of copper-based monolayer graphene lubrication film, water-based GO solution, Si wafer, Cu and Au access output ports, as shown in Fig. 4b.

These results show that interfacial lubrication can effectively improve the output of TENG. If deionized water is used as the interfacial lubricant between Si and copper-based monolayer graphene film, the current increases by an order of magnitude to 18 µA. Moreover, if the water-based graphene instead of water is used as a lubricant, the output current can be twice as high because of its better conductivity compared to water. Finally, under the action of interfacial lubrication, TENG can reach 155 µA peak current and maintain the output stability of 92–95% after 30,000 cycles. P–N type semiconductor is also one of the important methods to improve the tribovoltaic effect. Xu et al. reported a DC TENG with a p- and n-type doped semiconductor as the tribo-material, in which the current direction is determined by the built-in electric field in the p–n junction [73]. Zhang et al. displayed a high average power density direct current TENG with gallium nitride (GaN) and bismuth telluride (Bi_2_Te_3_) for the first time [71] (Fig. 4c). GaN, acting as a slider, is rubbed over Bi_2_Te_3_ at a contact pressure of 4 N, producing an open-circuit voltage of 40 V and a short-circuit current of 0.34 mA DC output. Contrary to previously reported work, the authors of this paper reported that the direction of current is not dominated by the built-in electric field between semiconductors, and that there are other causes for Bi_2_Te_3_ and GaN. By experiments, it is proved that the current of the junction always moves from Bi_2_Te_3_ to GaN, which indicates that the interface electric field comes from the CE process of the two tribo-layers, rather than the semiconductor's built-in electric field. In addition, the work by Li et al. [74] indicated that high operation frequency (*f* > 100 Hz) and large external load resistance (*R* > 10 MΩ) generate DC output, which proposed a strategy for harvesting extraordinary mechanical energy. Ren et al. reported a semiconductor-based DC TENG coupled with photovoltaic effect and tribovoltaic effect [72], as shown in Fig. 4d. This TENG contains P-Si and N-GaN as the tribo-layer, on the back of which there are electrodes as the output of the external circuit. When the silicon wafer moves onto the GaN, a dynamic p–n junction is formed to generate charge carriers on the interface. The direction of direct current is along the direction of built-in electric field. More importantly, ultraviolet light can regulate the output of the TENG. Based on the photovoltaic effect, when the ultraviolet light at 365 nm irradiates the interface between Si and GaN, the current and voltage of the TENG increase by 13 times and 4 times respectively, which has a great guiding role for the future development of the TENG.

#### Flexible Organic Semiconductors

**Fig. 5:**
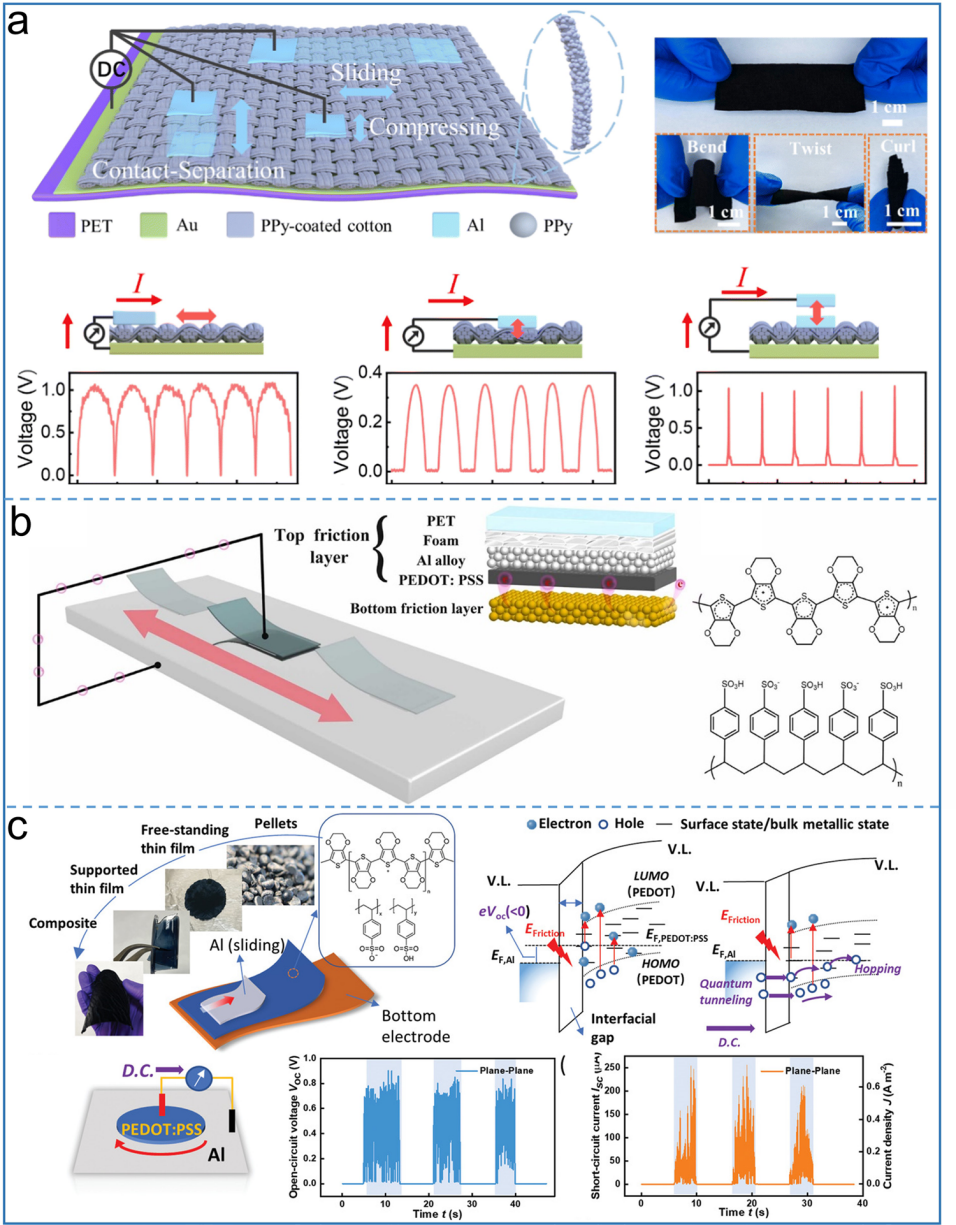
Schematic diagram of tribovoltaic effect based-DC-TENG using flexible semiconductor materials. Structure and working mechanism of **a** the flexible DC generator at different motion modes, sliding, compression and contact-separation mode TENG. Reproduced with permission [50]. Copyright 2022, Royal Society of Chemistry. **b** PEDOT: PSS/Al alloy DC TENG. Reproduced with permission [75]. Copyright 2022, Elsevier. **c** Flexible Al/PEDOT:PSS sliding contact system and corresponding open-circuit voltage and short-circuit current. Reproduced with permission [76]. Copyright 2021, Wiley–VCH

With development of wearable electronics, not only the output performance of TENG needs to be improved, but the flexibility of devices is also required. Organic semiconductor devices have attracted most attention for their flexibility and embedded ability in devices. Devices based on the sliding mode are known to have a lot of wear on semiconductors, although the current density is somewhat improved with tribovoltaic effect. Meng et al. demonstrated a durable flexible direct current TENG based on tribovoltaic effect in contact-separation mode [50], as shown in Fig. 5a. In order to form a flexible Schottky junction, polypyrrole (PPy) was chosen as an organic semiconductor due to its small bandgap structure and excellent flexibility. The authors explored the optimal output modes of TENG, including sliding, compression and contact-separation modes. The results show that those three modes produce DC output, but the contact separation mode has a better charge and peak current output than the other two modes, and reaches a 16.00 A m^−2^ current density and 98.72 mC m^−2^ charge density. The author used the non-contact mode to verify the influence of electrostatic induction on the output. As expected, the output in the non-contact mode was much smaller than that in the contact separation mode. Although this variable condition could not be the same as that in the contact mode, it was proved that the electrostatic charge was negligible and the tribovoltaic effect played a dominant role in this work. Similarly, a metal–semiconductor TENG demonstrated by You et al. [75] with ultrahigh short-circuit current is shown in Fig. 5b, because the metal material with an appropriate work function rubbed against the flexible PEDOT:PSS. The authors proved that a larger work function difference between the metal and semiconductor produces a better output performance. The work function of the two materials is the basis that determines the electron direction. If the work function of the friction electrode is greater than that of the semiconductor, electrons will flow from the semiconductor to the friction electrode. The experimental results show that the TENG output is also affected by the pressure, sliding speed, and device size, and it can achieve the optimal voltage output of 1 V and current output 309 µA under certain parameters. Thanks to its flexibility, high electric conductivity and low bandgap, PEDOT:PSS is again selected as a tribo-material of flexible Schottky TENG for organic semiconductors to produce DC output, which was proposed by Liu et al. [76]. In order to demonstrate the basic mechanism of metal-conductive polymers during contact, Al probe is used to slide over PEDOT:PSS to produce a DC output, as shown in Fig. 5c. The authors have confirmed that the current and polarity of the sliding Schottky junction are dependent on the interfacial electric field compared to the compression type Schottky barrier. With long-chain polymer concept, flexible Schottky DC generator demonstrates excellent performance. Under single contact geometry, the current density can reach up 20 A m^–2^. Metal/conducting polymer devices have lower internal resistance than traditional inorganic devices, generating peak power density at low resistance, which is an advantage of semiconductor based TENG.

### Multiple Phase Coupling DC-TENG

**Fig. 6:**
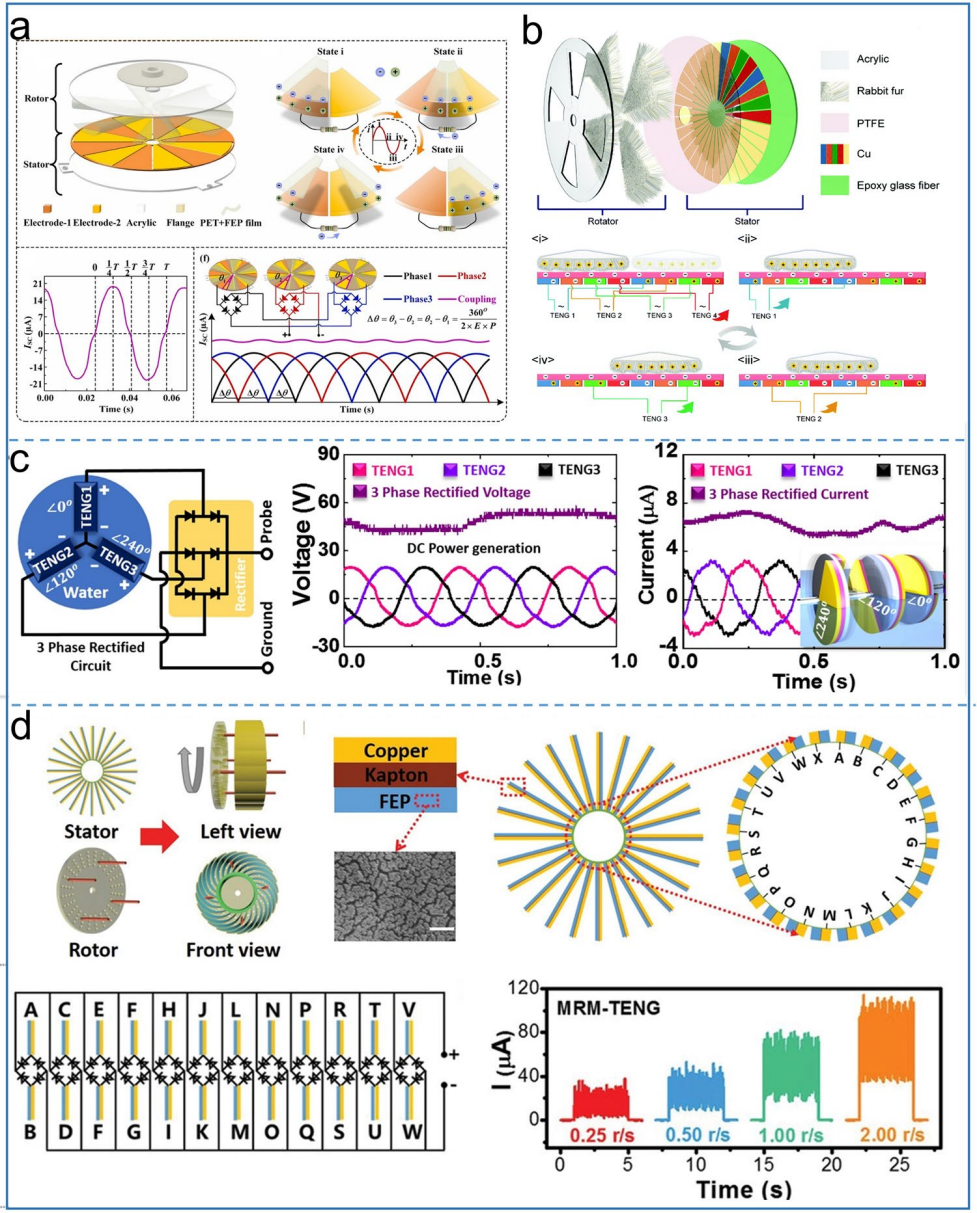
Phase control DC-TENG. **a** Structure, working mechanism and output characteristic of soft-contact mode DC-TENG. Reproduced with permission [55]. Copyright 2022, Elsevier. **b** Schematic diagram of rotating multi-phase TENG. Reproduced with permission [77]. Copyright 2021, Royal Society of Chemistry. **c** Working principle of Water-based DC-TENG. Reproduced with permission [56]. Copyright 2018, Elsevier. **d** Structure and output curve with different speeds of parallel multiple rectifier TENG. Reproduced with permission [44]. Copyright 2020, Wiley–VCH

The crest factor is the ratio of the peak value of pulse output to the effective value, which is one of the important indicators to measure the output performance of the TENG. The closer this value is to 1, the better the performance of the TENG will be. However, the crest value of traditional AC output TENG can be up to 6, which greatly limits the TENG as an energy source for practical applications. The multiphase coupling strategy not only reduces the crest factor to approach 1, but also solves the electric loss in the simple parallel output state of traditional multi-unit TENG, which has become one of the hot topics in DC TENG nowadays. A novel soft contact design was first reported by Jiang et al. [55], as shown in Fig. 6a, where multi-phase TENG consists of three independent rotating devices with a certain angle difference. For a single-phase TENG, the flexible FEP film first contacts electrode 1, and the two tribo-layers have the same amount of dissimilar charges. Under the action of external force, the FEP film rotates and completely coincides with electrode 2. At this time, electrons flow from electrode 2 to electrode 1, generating output in the external circuit. Subsequently, the authors demonstrate the basic principle of multiphase TENG by three TENG units and six pairs of electrodes. The Angle difference between the stator positions of adjacent single-phase can be defined as:

1 $$ \Delta \theta = \theta_{3} - \theta_{2} = \theta_{2} - \theta_{1 } $$

Δ*θ* is determined by the number of phase (*P*) and electrode pairs (*E*):

2 $$ \Delta \theta = \frac{{360^{^\circ } }}{2 \times P \times E} $$

The experiment shows that the crest factor decreases from 1.31 to 1.05 with the increase of the number of phases and electrode pairs, achieving continuous DC output. This TENG achieves an ultra-high output current density of 3021 μA m^−2^, 6.93% higher than that of a single-phase TENG without phase difference. The degree of contact is one of the important factors affecting the output performance of the TENG. This elastic FEP film design not only reduces the friction and torque forces, but also improves the output performance relative to the hard friction.

Chen et al. invented the soft contact multiphase DC TENG based on the friction layer of rabbit fur, as displayed in Fig. 6b [77]. Based on the electrode misalignment and circuit connection strategies, this TENG achieves ultra-low crest factor of 1.05 and ultra-high average power compared with traditional single-phase devices. The TENG consists of two parts: the rotor and the stator. The lower part of the stator consists of 32 independently separate electrodes constructed by printed circuit board (PCB) technology, which was covered by PTFE film. The rotor is fabricated by four fan-shaped friction layers of rabbit fur, each of which covers four electrode regions. The tribo-layer area of the traditional free-standing mode is the same as that of an independent electrode. The difference for this TENG is that a pair of electrodes separated by three electrodes is an energy output unit, which has two electrodes of the same color in the diagram. Due to the triboelectrification effect, in the contact process of PTFE and rabbit fur, rabbit fur is positively charged, while PTFE is negatively charged, when the rotor rotates an electrode region. In order to achieve potential balance, electrons flow between the same color electrodes. Thereby, the external circuit produces output. The author investigates the effect of a number of phases on the output performance. When adjusting phase from 1 to 9, the crest factor, charge transfer and RMS current are greatly improved. This indicates that the fabrication of super multiphase output structure is a future trend for multiphase coupling DC-TENG. At the same time, it will also face complex manufacture process. Ryu et al. designed a novel asymmetrical DC-TENG based regularly shifted phases [62]. The rotor part of the TENG is composed of the same number of alternately arranged PTFE and nylon. The PTFE layer is attached to the top of scattered electrodes as the stator part. Dividing the entire circular area into several small areas of the same size has proven to be a method to change the TENG output. Based on this design, the value of the current generated by each of the two electrodes is the same, but each current has a certain phase difference. The AC output of the multiple phases can be formed a constant DC output through full wave rectification. The success of this multiphase coupling brings the TENG's crest factor to 1.26, along with high output of 3.6 mA m^−2^ current density and 4.9 W m^−2^ power density.

Considering durable properties of the tribo-materials, Kim et al. proposed a phase-controlled water triboelectrification DC TENG [56], as shown in Fig. 6c. Take the individual TENG as an example, the authors constructed a pair of PTFE attached to the electrode on both sides of the acrylic disc with a phase difference of 180°. Half of the disk is always in water, and because of this clever design, two electric potential sources are generated on both side of the disk during a single rotation. With n disks and 360°/n phase difference, the multiphase TENG has better output performance. The authors fabricated a 3 disks with 120° phase difference TENG, realizing a 3 power output source, which can light LEDs without any flickering. Wang et al. reported a multiple phases and multiple groups coupling low crest factor (1.08) direct current TENG with cylindrical structure design [78]. 3P3G is taken as an example to analyze the mechanism, that is, the multiphase TENG contains three TENG units, and each TENG contains three groups. In the process of rotor rotation, the FEP rubs with the copper electrode, and the external circuit generates AC signals. Since the contact positions of FEP and electrode are different in the initial stage, each TENG unit has a different AC phase. Finally, rectifier bridges are used to integrate the three phase outputs together, resulting in a continuous high-performance DC output. The rotating contact-separation mode proved to be an effective measure for increasing TENG lifetime by Li et al. [44], and benefiting from the phase difference of each TENG, multiple TENGs are connected in parallel. As a result, an ultra-low crest factor is achieved (Fig. 6d). The stator part of the TENG consists of 24 copper electrodes and FEP films, and the kapton serves as supporting layer located between the electrodes and FEP films. Each two adjacent electrodes serve as an output unit, and the electrode and FEP realizes periodic contact-separation under the rotor push rod. Due to different sites generated by each output TENG, multiple AC outputs with phase differences are formed at the output end. Subsequently, the author proves the superiority of multiphase TENG in parallel through different connection methods. Based on the conventional connection mode, multiple electrode connections achieve high crest factor AC output without a rectifier bridge. When each TENG unit is connected with a rectifier bridge and these outputs are connected together, a continuous DC output with a higher current density is formed compared with only one rectifier bridge connected to the output end of the electrode. It should be noted that although the rectifier bridges might be used in multiphase coupling DC-TENG, its output has much lower crest factor and becomes continuous DC output.

### DC-TENG Using the Mechanical Delay Switch

**Fig. 7:**
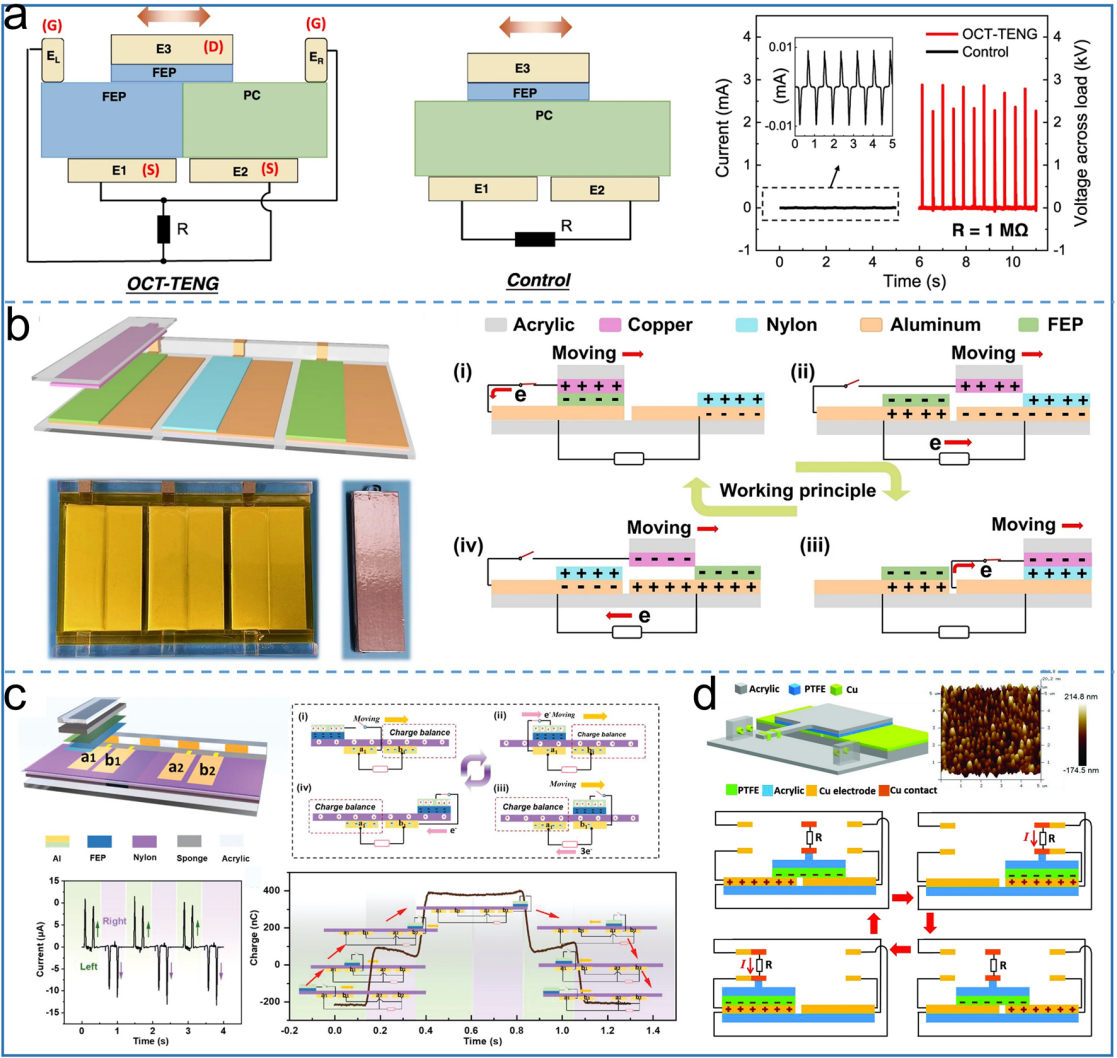
Mechanical switch based DC-TENG. **a** Comparison of device and output performance between opposite-charge enhanced transistor-like TENG and traditional sliding TENG. Reproduced with permission [51]. Copyright 2021, Springer Nature. **b** Schematic diagram and working mechanism of primary cell structure TENG. Reproduced with permission [79]. Copyright 2022, Springer Nature. **c** 3D structure diagram and working mechanism of mechanical time-delay switch and charge space accumulation DC-TENG. Reproduced with permission [52]. Copyright 2022, Wiley–VCH. **d** Structure diagram of unidirectional switch DC-TENG. Reproduced with permission [80]. Copyright 2018, Wiley–VCH

When two electrodes contact at a certain moment during the operation, the current becomes a single-channel DC output using mechanical delay switch, which reduces the energy loss caused by the rectifier circuit used in a traditional TENG. Inspired by the working principle of transistors, Wu et al. designed a TENG based on the opposite charge enhancement effect [51], as shown in Fig. 7a. The stator part of the TENG contains FEP and PC tribo-layers with opposite polarity, and there are two electrodes E1 and E2 below them. Different from the traditional TENG, there is a switch electrode in the upper left of FEP and the upper right of PC to form a transistor like structure, and the slider is the FEP film and the E3 electrode is located above it. When the slider sides from left to right side, that is, the time before the E3 makes contact with ER, the transistor is off and the switch is on. Similarly, the transistor is on and only E3 is in contact with El, making the charge transfer from the source E1 or E2 to the drain E3. At this time, there is an output impedance close to 0, thus forming an ultra-high charge transfer. For this design, the TENG achieves a current of 2.7 mA at a load of 1 MΩ, 300 times that of the traditional TENG. When the load is 12 ohms, an instantaneous power density of 10 mW m^−2^ is achieved and 100 W commercial bulbs can be lighted. In 2022, Fu et al. delivered an ultra-durable DC TENG based on primary Cell mechanism using motion mechanical switch [79]. The device structure and working mechanism is shown in the Fig. 7b. The mechanical switch of this device is designed at one end of electrodes which are covered with alternating FEP and nylon film and the copper electrode serves as the slider. When the sliding block of copper electrode is in nylon film or FEP film, the switch is closed. The slider with negative charges moves onto the next blank electrode, and the switch between copper foil and aluminium electrode on the back of the PA film is open. As electrons are transferred between the blank electrode and the electrode covered by the tribo-layers, the external circuit produces a DC output. Similarly, Du et al. developed a double dielectric tribo-layers with space accumulation effect based DC-TENG [52], as shown in Fig. 7c. Under the mechanical delay switch, the TENG finally realizes unidirectional DC output characteristics. Thanks to the space charge accumulation effect, the friction layer blank region without the back electrode enables the TENG to achieve an average power density of 4.2 W m^−2^ and maintain a mechanical stability of 92% after 120,000 cycles. More importantly, in the rotating state, the TENG can supply power to 24 hygro-thermographs in parallel, which demonstrates superb performance of the mechanical time-delay switch TENG. The unidirectional switch DC-TENG is proposed by Qin et al. [80], with the help of PMC, realizing 75.8% energy storage efficiency under theoretical calculation, as shown in Fig. 7d. Using a freestanding mode, the stator of the TENG consists of two electrodes and four contact electrodes attached to the electrodes on both sides. The slider is PTFE attached to the bottom of the acrylic substrate and has two moving electrodes on its epitaxial. Only when the slider moves to the far left or far right of the stator, the two point electrodes contact and the switch closes, meanwhile the charge transfer reaches the maximum, and the pulse output in the same direction is generated.

**Fig. 8:**
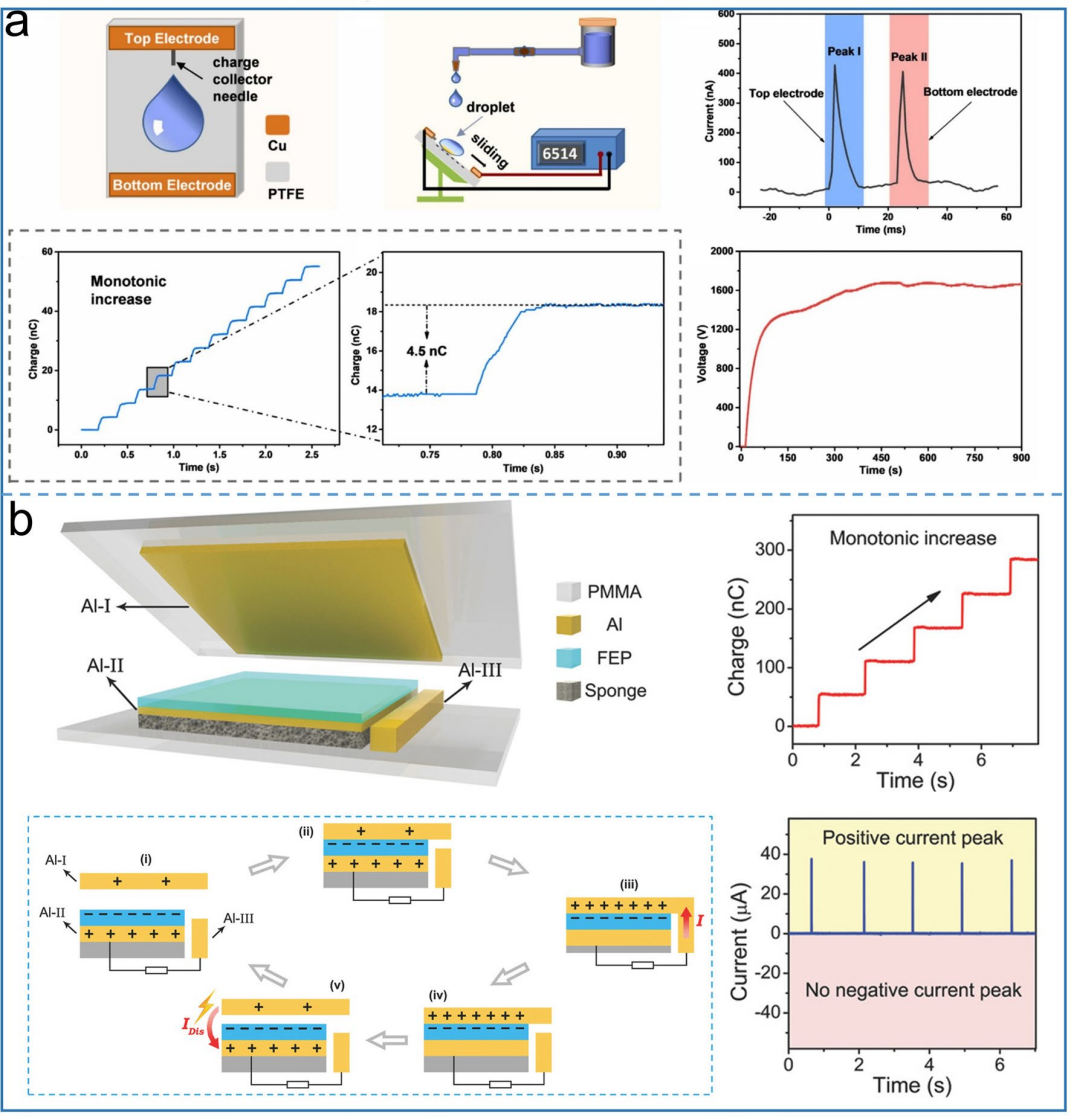
Schematic diagram with mechanical switch strategy of **a** droplet-based electricity generator, Reproduced with permission [22]. Copyright 2021, Elsevier. and **b** contact-separation mode TENG. Reproduced with permission [63]. Copyright 2018, Wiley–VCH

Based on the solid–liquid structure, Song et al. reported a water droplet based high voltage direct current TENG [22], and Fig. 8a shows the water droplet as the charge shuttle, which can deliver positive charges to the top electrode and negative charge to the bottom electrode. Different from the previously reported water droplet structure [81], the bottom electrode of this TENG is very narrow in width and located above PTFE. Only tens of circulating water drops are needed to achieve the optimal output, which also has better triboelectrification property than that of the reported TENG [81], which requires tens of thousands of drops to reach the maximum output. As soon as a drop falls, the top and bottom electrodes act as a reservoir, accumulating charge continuously. Each drop can transfer 4.5 nC charges, and generate 1600 V voltage that can light up 400 LEDs. Another important work in contact-separation mode TENG with DC output is reported by Luo et al. [63] as shown in Fig. 8b, The TENG contains three Al electrodes, named Al-III, and Al-II is placed nearby Al-I as a prominent design of the DC output, connecting Al-III with Al-II for the output of the external circuit. Thanks to the synergistic action of the foam buffering and Al-III electrode, Al-I and Al-III are cut off earlier than the separation of Al-I and PTFE during the operation to the separation stage, which prevents the charge from returning to the original direction under the action of potential difference. Thereby, the gap between Al-I and Al-II generates a high electric field, ionizing the air to form an ion channel that carries back a positive charge from Al-I to Al-II, thus creating unidirectional current output under both contact and separation states. In this way, the TENG achieves a peak power of 1.83 mW and a peak current output of 30 μA with a relatively low matched resistance (2 MΩ).

### Air Discharge

In addition to the above DC generation strategies, the mode by combining air discharge with the triboelectrification effect is considered to be an effective output way to generate high surface charge density, average power density, and output voltage compared with traditional output modes based on triboelectrification effect and electrostatic induction. A micron or millimeter-scale air gap between the dielectric tribo-layer and the metal electrode is a necessary condition to generate a high electric field that ionizes the air to form an air channel. According to different material properties of the two tribo-layers, the air-discharge TENG is divided into two categories: metal-dielectric mode and dielectric-dielectric mode. And the structure, mechanism and output performance of the devices are compared.

#### Metal-Dielectric DC-TENG

Liu et al. first invented the TENG that can produce continuous DC output based on the triboelectric effect and air breakdown [64]. The TENG contains two electrodes: one is a friction electrode (FE) as a friction layer of the TENG, and the other is a charge collecting electrode (CCE) attached to the side of the slider acrylic, which has a certain air gap to the stator PTFE. As FE is in contact with the stator, FE performs positively while PTFE carries the same amount of negative charges. Under the action of external forces, the slider will move from left to right. And benefiting from the intrinsic characteristics of the electret in PTFE, negative charges accumulate in PTFE surface, forming a strong electric field between PTFE and CCE. As long as the electric field strength exceeds 3 kV mm^−1^, the air is ionized and the charge is transferred in the air gap, producing a DC output in the external circuit. The authors prove that only there is output in the outer circuit in one sliding direction. Because the surface of PTFE is continuously covered by FE when the slider moves towards CCE direction, and the electric field cannot reach the threshold of air breakdown. Although the output is improved to some extent, reaching 430 µC m^−2^ and the crest factor is close to 1, the planar friction layer wastes some space efficiency, for the output charge of such TENG is related to its sliding distance.

**Fig. 9:**
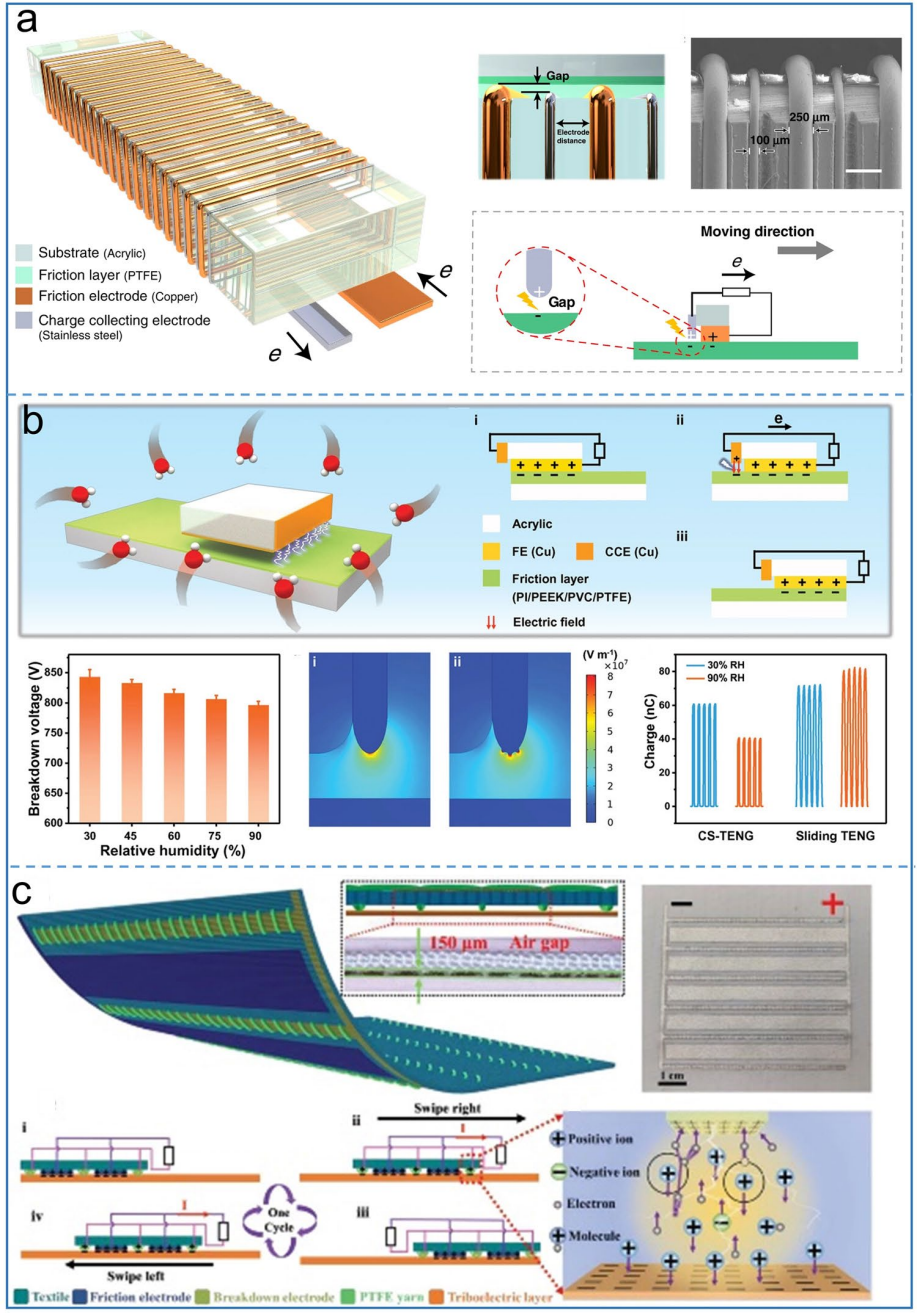
Metal-dielectric air-discharge DC-TENG. Improving output performance of **a** patterned electrodes units design, Reproduced with permission [53]. Copyright 2020, Springer Nature. **b** High humidity, Reproduced with permission [82]. Copyright 2022, Wiley–VCH. **c** Flexible tribo-materials structure. Reproduced with permission [83]. Copyright 2022, Wiley–VCH

Subsequently, Zhao et al. reported that the pattern electrode structure achieved a surface charge density of 5.4 mC m^−2^ [53], 2 times higher than that of the traditional AC TENG. As shown in Fig. 9a, 250 µm wide copper wire and 150 µm wide steel wire are wound on an acrylic substrate as FE and CCE respectively, where the distance between each pair of FE is 1000 µm. There is an air gap between CCE and PTFE while FE and PTFE are in close contact. Through the electrode microstructure strategy, the authors propose that the limiting factor of the surface charge density of the metal dielectric DC TENG can be described as:

3 $$ \sigma_{{\text{DC - TENG}}} = k \times {\text{min}}\left( {\sigma_{{{\text{triboelectrification}}}} ,\sigma_{{{\text{c,electrostatic }}\,{\text{breakdown}}}} } \right) $$

The integration of more electrode units’ k in the slider of the same area will result in higher surface charge density. Based on basic normalized properties of the material including charge density, friction coefficient, electric field and stability, PVC is regarded as the most suitable friction material for the microstructure of DC TENG [84]. By designing the pattern structure to 50 units, Wang et al. achieved a surface charge density of 8.80 mC m^−2^ that exceeds all kinds of previous TENGs. However, the output voltage is not in proportion to charge density due to its multichannel output mode. The researchers explored a number of strategies to improve the output performance of the metal-dielectric mode TENG, including interface lubrication, vacuum environment and gas atmosphere. Interface lubrication is considered to be an effective way to improve the maximum power output and durability of the DC-TENG due to the tiny gap between the two friction layers which limits the interface breakdown [85]. The experiment confirmed that high temperature and low pressure environment is more conducive to the occurrence of air breakdown, and achieved the improvement of power density compared with that in normal atmospheric pressure and room temperature conditions [86]. Similarly, the current output of the TENG can be increased threefold when oxygen is used instead of air as the device's operating environment [87]. High humidity is generally considered to be a negative factor for TENG output performance because it affects the electrification characteristics of the tribo-layer. Recently, Liu et al. reported that humidity as a positive factor improved the performance of the DC-TENG [82] as shown in Fig. 9b. By integrating 20 electrode units, the charge density reached 2.97 mC m^−2^ even at 90 percent humidity. Compared with sliding friction, rolling friction can reduce material wear and improve energy conversion efficiency which is considered as a method to improve the stability of DC-TENG. Gao et al. designed a metal-dielectric DC-TENG containing a support structure and a variable number of rolling friction electrodes, which achieved 96% stability in 90,000 cycles [88]. By coupling triboelectrification and air discharge effects, Shan et al. designed an inverting TENG from DC to AC with opposite electrical polarity properties (kapton and nylon) as tribo- layer, which is also used as a self-powered sensor [38]. A symmetry output mechanism for the TENG, from AC to DC, and from DC to AC, or even hybrid DC and AC in one TENG is realized for the first time. Considering the lightness, flexibility and power supply of wearable clothing in practical applications, the flexible fabric TENG has been mentioned in previous reports. However, it is a challenge for the development of fabric TENG because the AC TENG requires rectification for electronic devices. Cheng et al. reported a fabric based DC TENG [83], whose output maintains a record in the current fabric TENG (Fig. 9c). By combining with efficient energy management (EEM), the TENG successfully demonstrated the ability to power popular electronics and its superiority as a wearable fabric device. The TENG contains a friction electrode, a breakdown electrode and a PTFE yarn. In particular, air gap between the breakdown electrode and the friction layer can be adjusted by PTFE yarn, which surrounds the breakdown electrode and can be sewn on ordinary clothing. Under the action of EEM, the matching impedance of the TENG is reduced from 200 to 1.6 MΩ, maintaining a high average power of 0.365 mW for long-distance wireless transmission, which provides a basic guarantee for wild survival through the wearable fabric TENG.

#### Dielectric–Dielectric DC-TENG

**Fig. 10:**
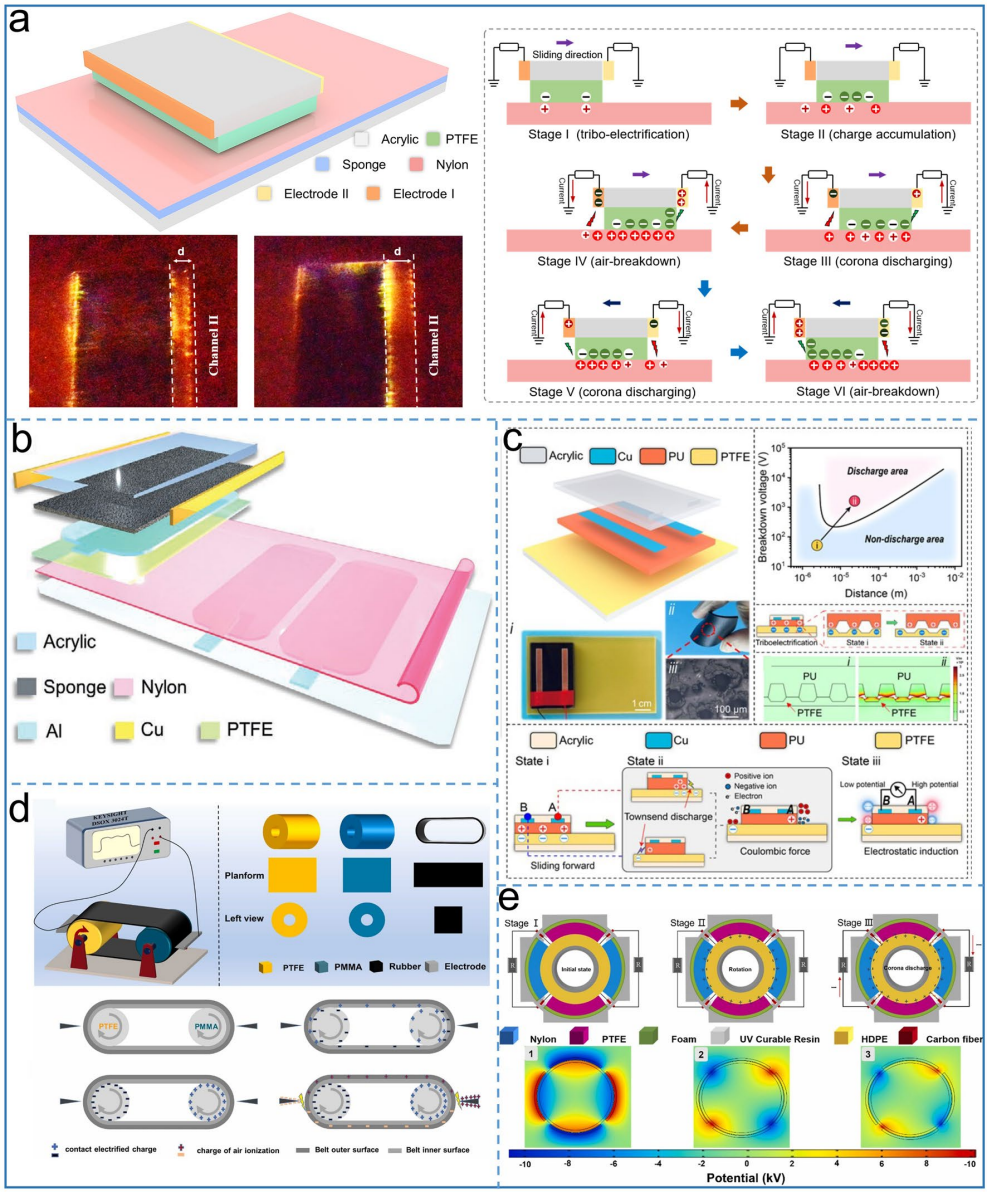
Dielectric-dielectric air-discharge DC-TENG. Schematic diagram and working mechanism of **a** bidirectional and double-channel corona discharge TENG, Reproduced with permission [54]. Copyright 2022, Wiley–VCH. **b** DC and AC double output TENG, Reproduced with permission [89]. Copyright 2022, Wiley–VCH. **c** AC/DC convertible output DC-TENG, Reproduced with permission [91]. Copyright 2022, Wiley–VCH **d** Rolling-mode DC-TENG, Reproduced with permission [92]. Copyright 2022, Elsevier. **e** Synergistically enhanced dual-breakdown DC-TENG. Reproduced with permission [93]. Copyright 2022, Elsevier

Although the surface charge density of metal-dielectric DC TENG based on air discharge has achieved a breakthrough achievement, the development of the discharge type TENG is still a challenge due to its low average power density and the complex preparation process and material wear of metal-dielectric mode TENG. Based on the triboelectrification effect and corona discharge [54], Shan et al. designed a dielectric- dielectric DC TENG as shown in Fig. 10a. Through the electrode structure on both sides of the substrate, a bi-directional dual-output channel DC TENG is realized. In the case of one of the left electrode, a bidirectional DC output is formed in the external circuit when the slider slides periodically left and right. It is confirmed by theories and experiments that a large amount of negative charges accumulates on the nylon surface when the slider moves rightward. As long as a strong electric field is formed between the nylon film and the left electrode over 3 kV mm^−1^, the left electrode produces a positive output. When the slider moves from the right to the left, a large number of negative charges accumulate on the PTFE surface and meanwhile corona discharge forms between the left edge of PTFE and left electrode. Thereby, the external circuit has a negative output. In addition, the spark is captured by a high-speed camera between the electrode and the tribo-layer, even if the electrode is placed at a distance of 10 mm, the discharge path can be clearly seen. By combining the space charge accumulation effect with local discharge [89], He et al. successfully proposed a novel DC and AC dual mode TENG coupling based on electrostatic induction and air breakdown effect as shown in Fig. 10b. Charge dissipation in air is inherent properties of materials. The introduction of corona discharge output channel reduces the charge loss and improves the energy utilization efficiency compared with the traditional single output mode TENG. In this way, the dual-output channel TENG achieves 5.74 W m^–2^ Hz^–1^ and powers high-power devices such as wireless optical sensors and electronic tablet. Considering the flexible and wearable electronics, flexible TENG has become a development trend. Shan et al. reported a dual DC output mode for TENG with flexible lint-free cloth [90]. The electrodes on the slider and stator are called as corona discharge electrodes and electrostatic induction electrodes respectively, which serve to collect corona discharge and collect trapped charge (not involved in corona discharge) on the surface of the lint-free cloth. By effectively utilizing triboelectric charge, the total output of two channels is greater than the single channel output, finally achieving a maximum output power density of 9.2 W m^−2^, which holds the highest record in the fabric TENG field. By coupling of triboelectrification, electrostatic induction and electrostatic discharge, Zeng et al. proposed a novel energy conversion mechanism to produce AC/DC convertible outputs TENG with wide material selectivity including polymers, fabrics, and semiconductors [91].

Different from the placement of the traditional DC-TENG electrodes, a pair of electrodes of TENG were placed on both sides of the back of a 2 mm PU film as the slider, and PTFE was used as the stator to be rubbed with the PU as shown in Fig. 10c. A large number of visible sparks can be observed in the air gap between tribo-layers. During the process of PU sliding on the PTFE layer, the air discharge forms a charge-transfer channel, generating a single-direction DC output. Moreover, the current value decreases by only 0.4% even if the load reaches up to 100 MΩ, indicating that this TENG is an excellent constant DC output source. Recently, Sun et al. [92] invented a rolling mode DC-TENG based on charge pump and air ionization, including a PTFE roller, PMMA roller, a rubber belt and two electrodes located on the side of the roller as shown in Fig. 10d. In this design, charges accumulate on the roller, and as long as the electrostatic field between the roller and the electrode is large enough to ionize air, some air ions are collected by the electrode while some weaken the electric field of the drum. The continuous movement of the conveyor belt produces a unidirectional charge flow between the left and right electrodes, forming a steady DC output. Current researches have proved that both durability and output performance of TENGs can be improved by the rolling design. Zheng et al. has invented a rolling DC TENG for cleaning indoor air pollution [93], which relies on a high voltage output of 27 kV. In order to collect triboelectric charge by corona discharge strategy to prevent the cancellation of positive and negative charges during the discharge, nylon and PTFE with high electron-affinity difference were used as polarizer for the stator (Fig. 10e). The high density polyethylene (HDPE) material with intermediate electric properties was attached to the rotor. As the rotor moves clockwise, the positive or negative charges accumulate along with a strong electric field. Subsequently, the charges are collected in the region where the electrode is located and the DC output is generated in the external circuit. HDPE as a tribo-material has better output of both the current and voltage relative to PET or Kapton in this work, because of similar electronegativity with PTFE. However, triboelectrification is poor during the friction process, which affects the overall output.

## Conclusions and Prospective

In recent years, the DC-TENG has been rapidly developed to become a promising branch of TENGs. Aiming to clearly sort out the types of DC-TENG and the limitations of output performance, we have made a review of DC-TENG based on the structure design, working mechanism and output performance as shown in Table 1. The current classification of DC-TENGs are divided into pulse current and constant current, which are derived from mechanical decay switch, tribovoltaic effect, and phase control, mechanical rectifier, air discharge, respectively.

**Table 1:** Comparison for output current *I*, voltage *U* and power density *P* of different types of DC-TENGs

Materials	I (µA)	*U* (V)	*P* (W m^−2^)	References
P-Si/N-Si	0.05	0.31		[73]
Au-PPy-TiO_2_	572.7	0.84	0.62	[94]
Steel-Si	0.25	0.65	0.0037	[49]
GaN-Si	0.154	3.35		[72]
Water-based GO	45	0.08	0.00543	[70]
Metal-Si	20	0.02	0.15 × 10^–3^	[95]
Bi2Te3-GaN	340	40	11.85	[71]
Au–Si	0.9		0.01	[74]
Al-PPy	1300	3.5	5.4	[50]
PEDOT:PSS-Al	200	0.8	0.67	[76]
PEDOT:PSS-Al	309	1	11.67 × 10^–3^	[75]
FEP-Cu	12	480	0.96	[58]
PMMA/PVC-Cloth	0.075	210		[96]
Kapton/Nylon-cloth	10.1	25,000	0.24	[57]
PTFE/Water	6	50		[56]
FEP/electrodes	97		2.5	[55]
FEP/Kapton	200	1100		[44]
Nylon/PTFE		380	4.9 (Average)	[62]
FEP/Copper	7.3	149.5		[78]
Rabbit fur-PTFE	21.28		1.11 (Average)	[77]
Cu-PTFE	2.5	720	0.01	[97]
Water/PTFE-Cu	0.45	1600		[22]
PTFE-Cu	17.5	350		[80]
FEP-Nylon	104.3	4500	4.2 (Average)	[52]
PVC-Al			25 × 10^–3^	[59]
FEP-Al	30	150	2.03	[63]
FEP/Nylon-Cu	56	2800	3.1	[79]
FEP/PC	2700		0.01	[51]
Kapton/Cu	150		82.25	[85]
Yarn-PTFE	40	4500		[98]
Yarn-PTFE	6.52	1229	0.18	[83]
PTFE/Nylon-Cu	1.9	460	0.095	[38]
PTFE-FE	7	500	0.20	[86]
PVC-Cu	2.1	260	0.95 W m^−2^ Hz^−1^	[82]
PTFE-Cu	65	33		[53]
PTFE-Cu	17	120		[64]
PI-Paper		600		[99]
PMMA/PTFE-Rubber	4.5	9000	7.9	[92]
Lint free cloth-PTFE	9.5	4800	9.8	[90]
PTFE-rubber	370	3200		[60]
PTFE-nylon	7	6200	5.74 Wm^–2^ Hz^–1^ (Average)	[89]
PTFE-nylon	3.8	6000	3 (Average)	[54]
PU-PTFE	3.9	3800	0.398 Wm^−2^ Hz^−1^ (Average)	[91]
BMF/PTFE-Rubber	197	1300	10.6	[100]
Nylon/PTFE-HDPE	2	27,000	2.83	[93]

Despite much attention to DC-TENG, there are still wide space for their development, and certain challenges still hinder their practical applications. More efforts are needed to break these limitations by scientists. (1) It is necessary to develop new DC output modes with portability, flexibility and simplicity of manufacture. In terms of phase control for DC-TENGs, continuous DC output requires multiple device units or multiple electrode units integrated in a TENG. Strict phase difference control increases the errors during device operation and reduces the stability of the power supply. (2) An excellent DC-TENG with overall high output performance is an essential condition for its commercial applications. Though this review does not focus on the development of power management system related with DC-TENG, it is worth discussing the attempts on output source and electric device development. Besides, power management is an indispensable part of TENG output, and power management circuit has achieved significant improvements in back-end resistance mismatches of AC-TENG. In particular, there is a lack of energy management for high voltage, low current (corona discharge) or high current, low voltage (tribovoltaic effect) problems of DC-TENGs. Next, the primary goal is to conduct point-to-point post-processing for various DC-TENGs to achieve high efficiency energy transfer. (3) TENG is regarded as a new type of sensor due to its sensitive signal, stable output and self-powered characteristics. The AC-TENG based on contact separation mode has made some achievements in self-powered sensors. However, as DC-TENG is in its infancy and lacks related sensor applications, development goes slowly and application fields are limited. Due to unique nature of the air breakdown DC-TENG, the current pulse width is dependent on the sliding distance, and displacement and velocity sensors can be designed and applied. Besides, an inverting TENG (from DC to AC) is reported, which can be integrated into self-powered sensors such as trigger switch and signal conversion. (4) Because of the nearly unlimited durability in all kinds of DC-TENGs, interfacial lubrication, dielectric-dielectric friction with thick dielectric material, flexible bent blade slider design, and non-contact mode should be employed to achieve the most promising wear cycles.
